# Er: YAG laser and superparamagnetic iron oxide nanoparticle-modified adhesive: A comparative study on antimicrobial efficacy and bond strength in caries-affected dentin

**DOI:** 10.12669/pjms.42.6.15668

**Published:** 2026-06

**Authors:** Mohammed A. Alrabiah, Fahad Alkhudhairy

**Affiliations:** 1 Mohammed A. Alrabiah, Department of Prosthetic Dental Science, College of Dentistry, King Saud University, Riyadh, Saudi Arabia; 2 Fahad Alkhudhairy, Restorative Dental Sciences Department, College of Dentistry, King Saud University, Riyadh, Saudi Arabia

**Keywords:** Er:YAG, Iron oxide nanoparticles, Streptococcus mutans, Shear bond, SEM

## Abstract

**Objective:**

Evaluating the efficacy of various cavity sterilants (CHX and Er: YAG) laser on the Streptococcus mutans (S. mutans) inhibition zone, resin tag length (RTL) and shear bond strength (SBS) of 5^th^ generation adhesive integrated with superparamagnetic iron oxide nanoparticles (SPIONs) bonded to cariously affected dentin (CAD).

**Methodology:**

The present in vitro study was conducted for four months, from 1^st^ July 2025 to 30^th^ October 2025. Forty-eight mandibular molars with ICDAS scores of 5 were included. CAD was retained and teeth were divided into four groups based on disinfection and adhesive type. Group-1A: CHX + Unmodified adhesive (UA), Group-1B: CHX+ SPIONs modified adhesive, Group-2A: Er: YAG laser+UA and Group-2B:Er: YAG laser+SPIONs modified adhesive. *S mutans* inhibition zone was identified using the agar diffusion test. RTL was assessed via SEM. SBS and failure patterns were analyzed using a Universal testing machine and a stereomicroscope, respectively. Data were analyzed via one-way ANOVA with post-hoc Tukey (*p* ≤ 0.05).

**Results:**

CHX and Er: YAG laser–treated *S. mutans* exhibited comparable inhibition zone thicknesses. Group-2B (Er: YAG laser + SPION-modified adhesive) showed the longest resin tags (20.55 ± 0.23 μm) and the highest SBS (8.23 ± 0.56 MPa). In contrast, Group-1A (CHX + unmodified adhesive) demonstrated the shortest resin tags (8.44 ± 0.52 μm) and the lowest bond strength (4.23 ± 0.43 MPa).

**Conclusion:**

Er: YAG laser appears to be an effective method for disinfecting CAD. Moreover, adhesives containing superparamagnetic iron oxide nanoparticles demonstrated superior performance, enhancing both RTL and SBS.

## INTRODUCTION

Dental caries remains a prevalent global health concern, involving bacterial invasion and demineralization of dental tissues. Streptococcus mutans (*S. mutans*), a key component of dental plaque, drives the initiation and progression of carious lesions.^1^ Carious dentin comprises two layers: the outer, irreversibly damaged caries-infected dentin and the inner, potentially remineralizable caries-affected dentin (CAD). Minimally invasive dentistry focuses on removing infected dentin while preserving the underlying CAD.^2^ Studies have reported that bonding to CAD results in reduced shear bond strength (SBS).^3^ Moreover, residual microbial colonies within the cavity may compromise restoration longevity by promoting secondary caries, highlighting the need for effective disinfection to enhance the tooth-restorative interface and improve clinical outcomes.^4^

Chlorhexidine (CHX) is a commonly used cavity disinfectant due to its broad-spectrum antimicrobial activity and ability to disrupt bacterial cell membranes. Its effectiveness against S. mutans and resistant strains has led to widespread use in caries management. Previous studies have reported conflicting results regarding its impact on the bond strength of dental adhesives to CAD.^5^,^6^ The literature provides insufficient data on CHX’s effect on resin tag length (RTL), highlighting the need for further research. Besides CHX, dental lasers, such as the Er:YAG laser, have gained interest as cavity disinfectants. Erbium lasers absorb well in water, enabling effective bactericidal action at low energy levels.^7^ These features position them as potential alternatives for traditional periodontal scaling, root planing, and cavity disinfection. Additionally, Er: YAG lasers remove the smear layer without causing thermal damage to the tooth.^8^ Despite these benefits, limited evidence exists on their impact on S. mutans survival, RTL, and adhesive bond strength of adhesive restoration to the CAD, necessitating further investigation.

The interface that exists between adhesive agents and dentin has been deemed the “Achilles heel” with respect to the longevity of the restoration. A resilient adhesive-dentin bond enhances the longevity of the restoration; however, its integrity is highly dependent on the formulation of the adhesives used.^9^,^10^ Recent investigations have concentrated on incorporating various nanoparticles (NPs) into dental adhesives to enhance their physical attributes and antibacterial efficacy.^9^ Magnetic nanoparticles, including superparamagnetic iron oxide nanoparticles (SPIONs) and their magnetically-assisted movement have provided solutions in biomedical sectors.^11^ SPIONs are biocompatible and respond only to a magnetic field, exhibiting superparamagnetic behavior. The implementation of magnetically enhanced dental adhesives driven by external magnetic force has been reported to assist dental adhesive infiltration into dentin, providing substantial validation as observed by Garcia and his team.^12^ Nonetheless, existing data remains limited and warrants further exploration.

The present study was designed under the assumption that incorporating SPIONs into a dental adhesive would alter resin tag length (RTL) and shear bond strength (SBS) compared with an unmodified adhesive under the same disinfection regimen (Er, YAG, or CHX). Furthermore, it was assumed that different cavity sterilization methods (Er, YAG, and CHX) would significantly affect the inhibition zones of S. mutans, RTL, and SBS of the two-step etch-and-rinse adhesive. Therefore, the objective of the present investigation was to examine the influence of various cavity disinfectants on *S. mutans* inhibition, resin tag formation, and bond strength of a fifth-generation SPION-enhanced adhesive applied to CAD.

## METHODOLOGY

A total of 48 human mandibular molars with ICDAS scores of five were recruited for this in vitro investigation. After ultrasonic debridement (Superior Instruments Co, New York, USA) of soft tissues and calculus, specimens were disinfected using chloramine trihydrate (Merck KGaA, Darmstadt, Germany) for 48 hours at 4°C The duration of the study was four months, from 1st July 2025 to 30th October 2025.

### Ethical Approval:

The in vitro study was approved by the ethical committee of King Saud University, CDRC# FC-289-25; dated June 10, 2025 and followed CRIS guidelines.

Each tooth was vertically embedded in auto polymerizing acrylic resin (Rapid Repair, Degu Dent GmbH, Hanau, Germany) within standardized polyvinyl molds. Selective caries removal was performed to eliminate infected dentin while preserving CAD, as determined by visual, tactile, and 0.5% basic fuchsin dye. The specimens were randomly assigned to two disinfection protocols (n = 24).^13^

### Integration of SPIONs into adhesive resins:

SPIONs (1 wt%) were incorporated into 5th generation Single Bond-2 adhesive (3M, Illinois, USA) using an ultrasonic probe homogenizer at 20 kHz and 70 W for 30 minute in an ice bath. Ultrasonication was intermittently halted every 10 minutes to prevent overheating and nanoparticle degradation, ensuring homogeneous dispersion.

### Group-1 (CHX):

CAD samples were treated with 2% CHX (Varni Corporation, Gujarat, India) for one minute, rinsed with water and air-dried.

### Group-2 (Er: YAG laser):

CAD specimens were treated using an Er: YAG laser (SAPPHIRE; Lightmed Corp, USA) with a wavelength of 2.94 μm and a pulse duration of 90 μs. The laser was operated at 1 W and 10 Hz, with the tip positioned perpendicular to the dentin surface at a fixed distance of 1mm. A controlled sweeping motion was used to irradiate a 4 × 4 mm area for one minute. The groups were further subcategorized into two subgroups based on the type of adhesive applied.

### Subgroup-A:

In this group, unmodified 5^th^ generation Single Bond-2 (3M, St. Paul, MN, USA) was used to build the composite. 37% phosphoric acid was applied, *followed by rinsing and drying*. Two layers of adhesive were applied, followed by a 10-sec curing process using a light-curing unit at 650 mW/cm^2^, with the tip held 2mm from the surface. Polyethylene tubes, with an internal diameter of 2 mm and a height of 2 mm, were securely affixed to the prepared dentin surfaces and subsequently filled with resin composite (Filtek P60, 3M ESPE, USA) before undergoing light polymerization.^14^

### Subgroup-B:

The SPIONs modified adhesive was administered in a manner identical to that employed in Group-1. Acid etching was subsequently succeeded by the application of the modified adhesive. The specimen was subsequently exposed to a magnetic field force for 60 secs via a cube-shaped magnet (maximum internal field 1.4 T, surface field 0.54 T; BX0X0X0-N52, K&J Magnetics, Pipersville, PA, USA). Each tooth was carefully positioned at the center of the magnet to guarantee that the magnetic force aligned with the axial direction of the dentinal tubules. Afterward, the magnet was removed, and the adhesive underwent photo-curing, and composite buildup was performed as in Group-A.

### Antibacterial susceptibility testing:

A pure culture of *S. mutans* was procured from ATCC 25175 (Microbilogics, St. Cloud, Minnesota, USA). The disk diffusion method was employed to assess the sensitivity of *S.mutans* to experimental disinfectants. Thirty ml of brain heart infusion (BHI) agar (Becton, Sparks, MD, Sigma-Aldrich) was dispensed into sterile glass Petri dishes. Three to five distinct colonies showcasing the same morphological characteristics were meticulously chosen from a blood agar plate culture and delicately transferred using a sterile loop into a tube filled with 5 mL of BHI broth, which was subsequently incubated at 37°C for 24 hours. A precise 50µL of the broth was promptly placed in the center of a dry BHI agar and evenly spread. Circular filter paper discs measuring 6mm in diameter were crafted from Whatman filter No. 1, arranged in a petri dish and sterilized in a hot air oven set at 160°C for two hours. Disks were infused with 20µL of each experimental disinfectant, utilizing three disks for each sterilant. The average of three measurements for the diameter of each inhibition zone around each disk was meticulously calculated. The test was conducted twice to ensure precision and reliability.^15^

### Resin tag length assessment via SEM:

The bonded teeth were bisected along the longitudinal axis utilizing a diamond saw. The cross-sectional surface was then treated with 50% phosphoric acid, followed by exposure to a 5% sodium hypochlorite (NaOCl) solution for 10minutes. Subsequent to dehydration in ethanolic solutions ranging from 70% to 100%, the specimens were air-dried and subsequently coated with gold through the process of sputter deposition. The samples were then analyzed using scanning electron microscopy (SEM) at different magnifications.^16^ The visual assessment of RTL was conducted utilizing established scoring criteria employing the four-step (0–3 scale) methodology as specified by Ferrari et al.^17^

### SBS examination and fracture pattern examination:

The specimens were subsequently analyzed for SBS employing the Universal Testing Machine (UTM) (Conten Industries, Inc, USA). Samples were subjected to a 5 kN load at a rate of 0.5 mm/min until the point of failure was reached. The failure pattern was assessed via a stereomicroscope and categorized into adhesive, admixed and cohesive classifications.

### Statistical analysis:

A one-way analysis of variance (ANOVA) followed by a post hoc test was used to assess RTL and SBS. (p<0.05) Statistical Package for the Social Sciences (SPSS) software version 20.0 (SPSS Inc., Chicago, Illinois, USA) was used for data analysis.

## RESULTS

### Inhibition zone:

The mean±SD of the inhibition zones of *S.mutans* after the application of various cavity disinfectants is shown in Table-I. The most extensive inhibition zone was exhibited by Group-2 (Er: YAG laser) (15.79±1.78 mm). Conversely, a restricted inhibition zone was exhibited by Group-1 (CHX) (6.24±1.13 mm). The analysis of intergroup comparisons revealed that both groups exhibited comparable inhibition zone thicknesses (p > 0.05).

**Table-I:** Inhibition zone of S. mutans after using different dentin surface sterilants.

Cavity disinfectants	Inhibition zone Mean ± SD (mm)	p-value!
Group-1: CHX	14.22±1.56 ^a^	< 0.05
Group-2: Er: YAG laser	15.79±1.78 ^a^	

!ANOVA Chlorhexidine (CHX), Erbium-doped Yttrium Aluminum Garnet (Er: YAG) laser. Different letters in superscript denote a statistically significant difference, Post Hoc Tukey (p<0.05).

### RTL Assessment:

**Fig.1:**
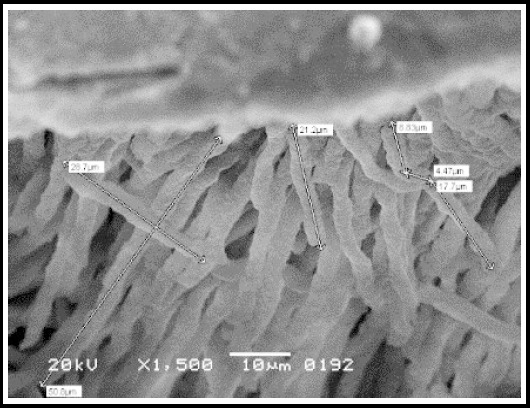
CAD disinfected with Er YAG laser with SPIONs modified adhesive.

The RTL of SPIONs modified 5^th^ generation adhesive bonded to CAD after applying various cavity sterilants is demonstrated in Table-II. Group-2B (Er: YAG laser+SPIONs modified adhesive) (20.55±0.23 μm) presented the longest resin tags. Whereas Group-1A (CHX+Unmodified adhesive) (8.44±0.52 μm) exhibited the shortest tag length. Intergroup comparative analysis showed that Group-1A, Group-1B (CHX+SPIONs modified adhesive (13.23±0.34 μm), Group-2A (Er: YAG laser + Unmodified adhesive) (16.32±0.31 μm), and Group-2B presented significantly different RTL amongst each other (p < 0.05) (Fig.1).

**Table-II:** RTL of SPIONs modified 5^th^ generation adhesive bonded to CAD after applying various cavity sterilants.

Tested groups	Mean ± SD (μm)	p-value!
Group-1A: CHX + Unmodified adhesive	8.44±0.52 ^d^	< 0.05
Group-1B: CHX + SPIONs modified adhesive	13.23±0.34 ^c^	
Group-2A: Er:YAG laser + Unmodified adhesive	16.32±0.31 ^b^	
Group-2B: Er: YAG laser + SPIONs modified adhesive	23.55±0.23 ^a^	

!ANOVA Chlorhexidine (CHX), Erbium-doped Yttrium Aluminum Garnet (Er: YAG) laser, Superparamagnetic iron oxide nanoparticles (SPIONs). The different superscript denotes a statistically significant difference, Post Hoc Tukey (p<0.05).

### SBS analysis:

The SBS of SPIONs modified 5th generation adhesive to CAD after various cavity sterilant is shown in Table-III. Group-2B (Er: YAG laser + SPIONs modified adhesive) (8.23±0.56 MPa) presented the highest bond SBS. Whereas Group-1A (CHX + UA) (4.23±0.43MPa) exhibited the lowest bond strength. Intergroup comparative analysis showed that Group-1A, Group-1B (CHX + SPIONs modified adhesive) (5.67±0.55 MPa), Group-2A (Er: YAG laser +UA) (7.03±0.78 MPa) and Group-2B presented significantly different bond strength outcomes (p<0.05)

**Table-III:** SBS of SPIONs modified 5^th^ generation adhesive to CAD after applying various cavity sterilants.

Tested groups	Mean ± SD (MPa)	p-value!
Group-1A: CHX + Unmodified adhesive	4.23±0.43 ^d^	< 0.05
Group-1B: CHX + SPIONs modified adhesive	5.67±0.55 ^c^	
Group-2A: Er:YAG laser + Unmodified adhesive	7.03±0.78 ^b^	
Group-2B: Er: YAG laser + SPIONs modified adhesive	8.23±0.56 ^a^	

!ANOVA Chlorhexidine (CHX), Erbium-doped Yttrium Aluminum Garnet (Er: YAG) laser, Superparamagnetic iron oxide nanoparticles (SPIONs). The different superscript denotes a statistically significant difference, Post Hoc Tukey (p<0.05).

### Fracture pattern analysis:

**Fig.2:**
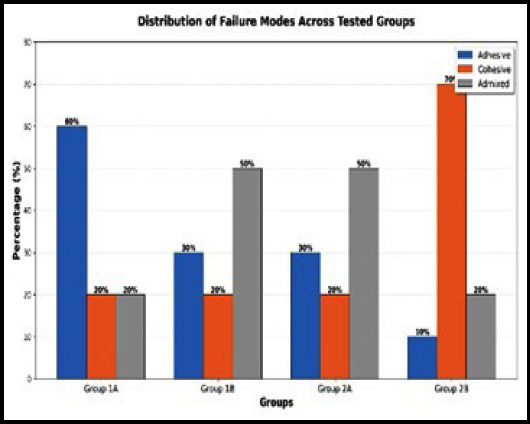
Distribution of Failure Mode Across Tested Groups.

Fig.2 delineates the allocation of failure modes (expressed as percentages) across the experimental cohorts. Group-2B exhibited the highest prevalence of cohesive failures. In contrast, adhesive failures were observed more frequently in Group-1A. Meanwhile, admixed failures were predominantly observed in Groups-1B and 2A.

## DISCUSSION

The present study was designed under the assumption that incorporating SPIONs into a dental adhesive would alter RTL and SBS when compared with an unmodified adhesive within the same disinfection regimen, i.e., Er, YAG, or CHX. Furthermore, it was also assumed that different cavity sterilization methods (Er, YAG and CHX) would significantly affect S.mutans inhibition zones, RTL and SBS of two step etch and rinse adhesive. Based on the outcomes, RTL and bond strength differ significantly within the same disinfectant group, thus accepting the primary hypothesis. Furthermore, it was observed that RTL and bond strength were significantly higher in the Er:YAG laser group than in the CHX group with comparable antimicrobial potency, thereby partially rejecting the second hypothesis.

Regarding the antimicrobial potency of the disinfectants used, CHX and the Er:YAG laser showed comparable efficacy against S. mutans. CHX is a positively charged, hydrophobic, lipophilic compound that engages with phospholipids and lipopolysaccharides in the bacterial cell membrane to facilitate cell entry.^2^ It dissociates and releases a positively charged CHX component through precipitation, culminating in cellular death.^13^ Er: YAG laser displayed satisfactory performance against S.mutans. Er: YAG laser coincides with the optimal absorption peak of water and has good bactericidal effects at low energy outputs.^18^ This aligns with in vitro analysis by Bohari and colleagues, who reported that Er: YAG reduces Streptococcus spp. rate by 90.2%.^19^ They supported these findings by noting that temperature rise from laser ablation affects micro-organism viability, altering cellular structure and eradicating bacteria involved in the carious process.^19^

Regarding RTL and bond strength, the group subjected to Er:YAG laser disinfection and subsequently restored with SPIONs exhibited markedly prolonged RTL and the highest SBS. The Er:YAG laser, compared with other laser modalities, has a relatively shallow energy penetration depth, resulting in diminished thermal injury to both periodontal and pulp tissues.^20^ This phenomenon can be elucidated by the observation that the Er: YAG laser, possessing a wavelength of 2.94 μm, has a specific absorption wavelength that corresponds to the primary constituents of dental hard tissues. The application of the Er:YAG laser for surface disinfection of dentin has the potential to enhance bond strength by effectively removing the smear layer and facilitating the opening of dentinal tubules, thereby establishing a more favorable surface for bonding material adhesion.^21^,^22^

Samples treated with CHX showed a significantly lower bond strength than that of the laser-treated group. Upon reviewing the indexed literature, it can be concluded that the effect of CHX as a cavity disinfectant on the SBS of dental adhesives remains a subject of debate. Certain studies have suggested that the initial bond strength is often influenced by CHX use, as it may precipitate on the dentin surface, disrupting the acid etching process and resin penetration and, in turn, hindering the development of the hybrid layer.^2^,^23^ Nevertheless, it plays a crucial role in maintaining long-term SBS by inhibiting matrix metalloproteinases (MMPs), which are responsible for the gradual breakdown of bonds.

The application of an adhesive infused with SPIONs, in conjunction with a magnetic field, has significantly facilitated deeper resin infiltration into dentin, thereby augmenting both bond strength and RTL. This finding aligns with the conclusions drawn from laboratory-based investigations performed by Garcia and associates.^12^ The enhancement in the density of resin tags can be attributed to the displacement of SPIONs within the adhesive into the dentin tubules, owing to their magnetically responsive characteristics.^12^ These magnetic nanoparticles proficiently guide the adhesive resin into the dentinal tubules, which possess diameters ranging from a mere one to two μm, in contrast to traditional micron-sized particles that face considerable difficulties in traversing these tubules.^12^

### Study limitations:

The research was conducted in vitro, which may limit its generalizability. Additionally, the study employed only a single adhesive, a single CHX concentration, and specific laser parameters. The magnet application time for nanoparticle infiltration was limited to 1 minute, which may have influenced the results. Consequently, further research is warranted to explore integrating “drug-loaded” magnetic nanoparticles into bonding agents, which could contribute to the development of innovative and effective approaches in conservative dentistry.

### Strengths of the study:

This study represents a pioneering comparison that simultaneously evaluates disinfection methods using SPION-modified adhesives. It introduces a dual-functionality innovation by combining antimicrobial and mechanical enhancements within a single system. The integration of SPIONs marks a novel application in fifth-generation adhesives.

### Contribution to Medical Literature:

The study provides an evidence-based protocol selection for clinicians, offering a rationale for adopting laser disinfection and nanoparticle-enhanced materials. It addresses the clinical challenge of bonding to compromised dentin substrates. Future investigations should optimize SPION concentrations, magnetic field strengths, and application durations to maximize tubular infiltration without compromising adhesive properties.

## CONCLUSION

Er: YAG laser is a viable option for disinfecting carious-affected dentin. Additionally, incorporating superparamagnetic iron oxide nanoparticles into the adhesive has demonstrated enhanced performance by increasing resin tag length and improving bond strength.

### Future directions:

Additionally, long-term aging studies incorporating thermocycling and simulated pulpal pressure are needed to validate bond durability. Research into drug-loaded magnetic nanoparticles that deliver antimicrobial or remineralizing agents within bonding systems could further advance bioactive restorative strategies for managing caries-affected dentin.

### Author’s Contribution:

**FAK and MAA:** Data collection, study design, manuscript writing, data analysis and final manuscript approval.

**FAK:** Is responsible and accountable for the accuracy and integrity of the present work.
